# The Analysis with Quantitative Indexes for Public’s Awareness of Radiation Knowledge in Taiwan

**DOI:** 10.3390/ijerph192013422

**Published:** 2022-10-17

**Authors:** Chen-Ju Feng, Yuan-Chun Lai, Shen-Hao Lee, Ke-Yu Lien, Ching-Yu Tseng, Ni-Shan Wu, Chiung-Ju Liang, Chin-Hui Wu, Shih-Ming Hsu

**Affiliations:** 1 Medical Physics and Radiation Measurements Laboratory, National Yang Ming Chiao Tung University, Taipei 112304, Taiwan; 2 Department of Biomedical Imaging and Radiological Sciences, National Yang Ming Chiao Tung University, Taipei 112304, Taiwan; 3 Department of Radiation Oncology, Changhua Christian Hospital, Changhua 500209, Taiwan; 4 Department of Medical Imaging and Radiological Sciences, Central Taiwan University of Science and Technology, Taichung 406053, Taiwan; 5 Department of Radiation Oncology, Chang Gung Memorial Hospital, Taoyuan 333423, Taiwan; 6 Chinese Society of Medical Physics, Taipei 110301, Taiwan; 7 Graduated Institute of Management, National Taiwan University of Science and Technology, Taipei 106335, Taiwan; 8 Department of Medical Imaging and Radiological Sciences, Tzu-Chi University of Science and Technology, Hualien 970302, Taiwan

**Keywords:** radiation awareness level, radiation education training, survey study, quantitative index

## Abstract

(1) Background: The purpose of this study was to evaluate the radiation awareness level of the public in Taiwan. (2) Methods: This study designed an online survey form to investigate the radiation awareness level with six topics: basic knowledge of radiation, environmental radiation, medical radiation, radiation protection, and university/corporate social responsibility. The score of respondents were converted into knowledge and responsibility indexes for the quantitative evaluation. Logistic regression was used to assess the correlation between the knowledge index and individual factors. Paired *t*-test was used to assess the significant difference in knowledge index between pre-training and post-training. (3) Results: The knowledge index of each job category reflected the proportion of radiation awareness of the job. The logistic regression result indicated that radiation-related people could get higher knowledge index. The paired *t*-test indicated that the knowledge index before and after class had significant differences in all question topics. (4) Conclusions: The public’s awareness of medical radiation was the topic that needed to be strengthened the most—the responses with high knowledge index significantly correlated with their experience in radiation education training or radiation-related jobs. It significantly increased the knowledge index of radiation if the public received radiation education training.

## 1. Introduction

Since W. Roentgen discovered the X-ray in 1895, radiation application has been widely used in energy, medicine, industry, etc. However, the inappropriate use of radiation can cause biological and environmental damage. The related organizations, such as the International Committee of Radiation Protection (ICRP), International Committee on Radiation Units (ICRU), and the International Atomic Energy Agency (IAEA), have responsibility for setting the guideline for radiation protection. Although radiation protection has been well established, general public may not know basic knowledge regarding radiation like food and drinking water they ingest contains natural radiation. Jointly the impact of international nuclear accidents [1] and the listing of radiation as a carcinogenic factor [2], the public has a negative cognition of radiation. The United Nations Foundation (UNF) has launched sustainable development goals (SDGs), which emphasize 17 issues such as environment, education, and human rights, enhance the implementation of social responsibility of cognitive equality, and then promote the balanced development of human society [3,4]. Whether it is university or corporate social responsibility [5,6], popularizing scientific knowledge to the public is an important issue. When faced with a controversial issue, if it exceeds the self-cognition or ability, most people will choose not to argue the issue, which may lead to general misconception [7]. Radiation has both positive and negative influences, so it must let the public realize the correct awareness of radiation to have the ability to distinguish the authenticity of relevant issues.

Medical radiation is the second largest source of radiation exposure after natural radiation, accounting for 20–50% of the personal radiation dose [8,9]. Medical radiation can be divided into three types: radiodiagnosis, radiotherapy, and nuclear medicine. According to the radiation protection guideline, the radiation instrument should be operated by radiation technicians. However, some invasive medical diagnoses need to be completed by the doctors and technicians together. These relevant staff may have the same risk of radiation exposure as the patient. Quinn et al. pointed out that the people without radiation education lacked clear awareness about radiation protection in UK [10]. Faggioni et al. investigated medical radiation awareness in medical students, radiography students, and radiology residents in Italy. It demonstrated that the performance of medical students’ radiation protection awareness was significantly lower than that of radiography students and radiology residents [11]. Qutbi et al. focused on the knowledge of radiation risk in the staff in a teaching hospital of Iraq, and they demonstrated the need for an increasing proportion of radiation protection training in radiology staff [12]. Naderi et al. pointed out that the patients needed to understand medical radiation, such as the type of radiological imaging instrument and the amount of radiation dose, which could reduce the health risk of patients caused by unnecessary radiation in Iran [13]. However, awareness assessment of radiation applications other than medical radiation is still a relatively lacking type of research. Through the comprehensive survey, the public’s awareness level of radiation protection in Taiwan can be used as a reference for radiation education that should be promoted in universities and corporate social responsibility. A better understanding of radiation could improve the public’s ability to distinguish the authenticity of radiation-related issues, protect themselves from the potential of radiation risk, and positively support the sustainable development of society.

The purpose of this study was to evaluate the radiation awareness level of the public in Taiwan. We surveyed to understand the public’s knowledge of various radiation application topics and their support for radiation education promotion. The quantitative index was established to analyze the influence of radiation awareness by the demographic characteristics and whether to receive radiation education training and engage in radiation-related jobs. This study discussed the significant improvement of personal radiation awareness by receiving radiation education training. In this way, the radiation education promotion was assessed as a part of the university or corporate social responsibility and the actual effect of improving the public’s awareness of radiation protection to achieve the goal of sustainable development.

## 2. Materials and Methods

### 2.1. Survey Design

The survey was designed in an online form (Google LLC, Mountain View, CA, USA) for this study. There were two sections in the survey. The first section was the personal information of survey respondents, including gender, age, education level, and job categories. Also, we investigated whether they had received radiation education and training and whether their job was related to radiation. The second section was the questions about radiation-related knowledge with six topics: basic knowledge of radiation, environmental radiation, medical radiation, radiation protection, and university/corporate social responsibility. The question descriptions within each topic were listed in the Supplementary File S1. All questions were developed through appropriate discussions with radiation professionals. Cronbach’s alpha value determined the reliability of the survey, and the alpha value was greater than 0.7 to evaluate the reliability of the survey [14].

### 2.2. Quantitative Index

In this study, the Likert scale was used to quantify the scores of each question [15]. The corresponding score was given according to the question of positive or negative statements. The total number of questions gave each respondent a raw score. Then it was converted into two percentile indexes: knowledge and responsibility.

The knowledge index was used to evaluate the awareness of radiation. It was converted from the score of five topics: basic knowledge of radiation, environmental radiation, medical radiation, radiation science and radiation protection, as demonstrated in Equation (1).

(1) $$\mathrm{Knowledge}\;\mathrm{index}=\frac{respondent\;score\;of\;the\;topic}{total\;score\;of\;the\;topic}\times 100\%$$

The total score for a single topic was 25. The knowledge index was summed by the result of each topic which was multiplied by the weighting factor (0.2 in each).

The responsibility index was converted from the topic of university/corporate social responsibility, as portrayed in Equation (2).

(2) $$\mathrm{Resposibility}\;\mathrm{index}=\frac{respondent\;score\;}{total\;score\;}\times 100\%$$

The total score for this topic was 25. The responsibility index evaluated the support for radiation education promotion in university/corporate social responsibility.

The subjects of this study were divided into two parts. The first part performed the statistical analysis for the knowledge index and responsibility index in public. The second part focused on the students who attended the radiation education training. It compared the knowledge index difference before and after the training to assess the benefits of radiation education training on the influence of the knowledge index.

### 2.3. Statistical Analysis

#### 2.3.1. Influence of Individual Factors on Knowledge Index

This study analyzed the influence of individual factors on the knowledge index. We used logistic regression to assess the correlation between the knowledge index and individual factors [16]: (1) demographic characteristics, including gender, age, occupation, and education level. (2) whether they have received radiation education training. (3) whether they engaged in a radiation-related job. Logistic regression used odds ratio (OR) as a comparison parameter. The higher the OR, the more relevant the individual factor was to the knowledge index. The median determined the high and low knowledge index [17]. In addition, this study used the Chi-square test [18] to assess the correlation between receiving radiation education training and engaging in radiation-related jobs. We also used a *t*-test [19] to analyze the significant difference in knowledge index of each age and education level who have received radiation education training and engaged in radiation-related jobs.

#### 2.3.2. Influence of Radiation Education Training on Knowledge Index

This study analyzed the survey respondents by the students who received radiation education training before and after class. The students receiving radiation education based on the requirement of their occupation and they were persons without radiation-related education before. The radiation education training provided the same training material to each student. We used a paired *t*-test to assess the significant difference in knowledge index between pre-training and post-training. The knowledge index was compared to each question topic: knowing radiation, environmental radiation, medical radiation, and radiation protection.

The statistical software used in this study was IBM SPSS Statistics 24 (IBM, Armonk, NY, USA), and the significant level was assumed at 0.05.

## 3. Results

### 3.1. Statistic Data of Survey Responses

**Figure 1:**
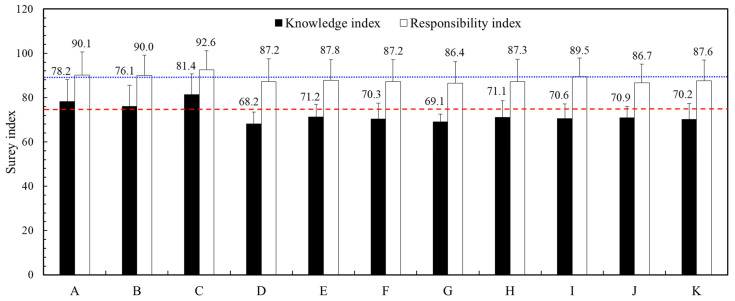
The knowledge and responsibility indexes corresponded to each job category. A: student, B: civil servant, C: board certification, D: financial industry, E: information technology, F: service and sale, G: communication and culture, H: manufacturing, I: construction, J: agriculture, forestry, fisheries and animal husbandry, K: freelance. The dash line portrays overall mean of knowledge and the dot line indicates overall mean of responsibility index.

The survey received 1104 respondents in this study. The Cronbach’s alpha values of the knowledge and responsibility indexes were 0.84 and 0.80, respectively. This result represented the reliability of the survey responses. The knowledge and responsibility indexes corresponding to the group characteristic of respondents are portrayed in Table 1. Of the male responses, 56.61% of all, 27.90% were at the age of 41 to 50 years, and 88.59% were at least a university education. The respondents who had received radiation education training were 27.26%, and those who engaged in radiation-relative jobs were 36.50%. For the knowledge index, the total mean value was 75.6. The knowledge index corresponded to five topics were 78.4 (basic knowledge of radiation), 76.5 (environmental radiation), 64.4 (medical radiation), 77.1 (radiation science) and 81.8 (radiation protection). For the responsibility index, the mean of all was 89.7. The responsibility index of the respondents who had received radiation education training was 94.3, and those who engaged in radiation-relative jobs were 93.9. Figure 1 indicated that the knowledge and responsibility indexes corresponded to eleven job categories. Among them, the knowledge index and responsibility index of student (78.2, 90.1), civil servant (76.1, 90.0), and board certification (81.4, 92.6) were higher than the overall mean.

### 3.2. Influence of Characteristic Factors on Knowledge Index

Table 2 demonstrated the logistic regression result of the knowledge index corresponded to demographic characteristics, whether receiving radiation education training or engaging in a radiation-related job. The respondents below 20 years were taken as the reference group, and the OR was 5.05 for the 20 to 30 years old. The respondents in secondary school and below were taken as the reference group, and the OR was 19.57 with the Ph.D. level. The OR of the knowledge index corresponded to receiving radiation education training and engaging in a radiation-related job were 14.52 and 10.70, respectively.

The Chi-square test result was significant (*p* < 0.001), indicating that receiving radiation education training was correlated with engaging in a radiation-related job. The *t*-test results of the knowledge index corresponded to receiving radiation education training and engaging in a radiation-related job within each age group were demonstrated in Table 3. There were significant differences in receiving radiation education training and engaging in radiation-related jobs across all age groups (*p* ≤ 0.001). The *t*-test results of the knowledge index corresponded to receiving radiation education training and engaging in a radiation-related job within each education level group were demonstrated in Table 4. There were significant differences in receiving radiation education training and engaging in radiation-related jobs across all education level groups (*p* ≤ 0.001). The knowledge index with received radiation education training was significantly higher than that without radiation education training.

### 3.3. Influence of Radiation Education Training on Knowledge Index

**Figure 2:**
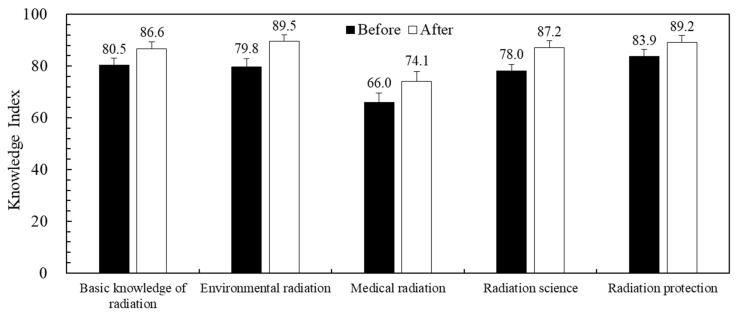
The knowledge index of the students before/after the radiation education training.

This study investigated 132 students of radiation education training. The results were from a subgroup of total respondents. The mean value of the knowledge index before and after the class was 77.6 and 85.6, and the mean value of the responsibility index were 91.9 and 94.3. Figure 2 demonstrated the changes in the knowledge index of each question topic for students receiving radiation education training before and after class. The paired *t*-test indicated that the knowledge index before and after class had significant differences (*p* < 0.001) in all question topics. The question topic with the most significant increase in the knowledge index was medical radiation (12.2% increase), and the smallest was radiation protection (6.4% increase). Also, the responsibility index of the students had significant differences (*p* = 0.033) before and after the class.

## 4. Discussion

In this study, we used a quantitative index to understand Taiwanese people’s awareness of radiation knowledge through a survey. We also analyzed the influence relation of knowledge index under different individual factors. Tinsley et al. pointed out that the adequate sample size of the survey should be ten times the number of questions [20]. The valid responses in this survey were 1104, so there was a sufficient sample size. In five question topics of radiation knowledge, the respondents had more awareness of radiation protection and basic knowledge of radiation. Most of the public awareness for medical radiation may be wrong. Therefore, the knowledge index of this topic was the lowest. The radiation from medical application caused the contribution second only to natural radiation for general public in Taiwan [21]. This result indicated that Taiwanese people lack knowledge in medical radiation application, so it was necessary to promote the correct radiation protection concept. The study in Vermont, USA stated that even though medical imaging was the primary source of ionization radiation, 80% of the population still underestimated the radiation exposure contribution by medical imaging [22].

The knowledge index of each job category reflected the proportion of radiation awareness of the job. The board certification had the most significant proportion of radiation jobs (67.4%, 223/331), so it had the highest knowledge index. The financial and communication and cultural industries were relatively alienated from radiation applications (3.8%, 2/52), so the knowledge index was relatively low. For ages, the knowledge index was higher than the total mean value for 21 to 30 years old. Then, it tended to decrease with the increasing age. The main reason was this age group with more people received radiation education (41.4%, 108/261).

For education level, the study in Nigeria assessed the public awareness of the ionizing radiation among students and lecturers in the college of education. It demonstrated that the correct awareness of students was lower than that of lecturers [23]. In this study, we divided the education level into four groups. The knowledge index in university, master’s, and Ph.D. were higher than the total mean value. The knowledge index of Ph.D. was the highest because the proportion of those who have received radiation education training was the largest in this group (60.9%, 28/46). This finding also means that the proportion of people who received radiation education training in each group had a potential preconception affecting on the knowledge index of this study. If it only discussed the condition of people who received radiation education training, the knowledge index tended to increase with the education level. The phenomenon also appeared in the condition of engaging in radiation-related jobs. However, age and education level reflected whether they had received radiation education training (84.5 vs. 72.3) or people engaged in radiation-related occupations (83.0 vs. 71.4), significantly impacting the knowledge index. Even the people with secondary or below education level, their knowledge index was higher than that of non-radiation-related people with Ph.D. levels after receiving radiation education training (81.4 vs. 78.4).

This study demonstrated that the knowledge index of students who received radiation education training increased significantly after class. At the same, the awareness of medical radiation was the topic that needed to be strengthened the most. Although the students knew that their jobs were radiation-related, their awareness of proper radiation knowledge was still insufficient. This study conducted the analysis to observe the simple relation between knowledge index and several individual factors. The general public could obtain radiation-related information or products through social media [24]. Therefore, the social responsibility of radiation knowledge promotion had the added value of increasing public awareness level of radiation. The responsibility index in this study reflected the public’s high recognition of radiation education promotion in universities and corporate social responsibility. These study results also demonstrated that the public was willing to participate in understanding scientific knowledge needs actively.

## 5. Conclusions

This study used a quantitative index to understand Taiwanese people’s awareness of radiation knowledge. The public’s awareness of medical radiation was the topic that needed to be strengthened the most —the responses with high knowledge index significantly correlated with their experience in radiation education training or radiation-related jobs. The people with secondary or below education levels, their knowledge index could be higher than that of non-radiation-related people with Ph.D. levels after receiving radiation education training. It significantly increased the knowledge index of radiation if the public received radiation education training. The responsibility of radiation education was not limited to the professionals. It should be promoted as basic social responsibility to improve the general public’s ability to distinguish radiation application information. Furthermore, the adoption in the university and corporate social responsibility could help people to know the right concepts of radiation for societal interest or adding value to business. This could make people realize the social responsibility of equality in scientific awareness and strengthen the fundamental integrity of sustainable development goals.

## Figures and Tables

**Table 1:** The results of two index corresponded to the demographic characteristics of the respondents participating in the survey.

	N	%	Knowledge Index (Mean ± SD)	Responsibility Index (Mean ± SD)
Total	1104	100	75.64 ± 9.7	89.7 ± 9.7
Gender				
Male	626	56.6	77.0 ± 9.6	90.9 ± 9.4
Female	478	43.4	73.9 ± 9.5	88.2 ± 9.9
Age				
Less than 21 years old	60	5.4	71.0 ± 6.6	86.5 ± 10.1
21–30 years old	261	23.6	80.2 ± 10.2	92.0 ± 9.7
31–40 years old	213	19.3	78.6 ± 10.4	91.1 ± 9.2
41–50 years old	308	28.0	73.3 ± 8.3	89.1 ± 9.7
51–60 years old	202	18.3	73.2 ± 8.4	88.3 ± 9.3
61 years old and above	60	5.4	70.1 ± 6.3	86.6 ± 9.3
Education				
Secondary school and below	126	11.4	71.9 ± 8.7	86.6 ± 10.1
Bachelor	549	49.7	76.5 ± 10.0	90.3 ± 9.8
Master	383	34.7	74.7 ± 9.0	89.3 ± 9.3
Ph.D.	46	4.2	83.7 ± 7.8	95.6 ± 7.1
Received radiation training				
Yes	301	27.3	84.5 ± 8.5	94.3 ± 8.3
No	803	72.7	72.3 ± 7.8	88.0 ± 9.6
Radiation related job				
Yes	403	36.5	83.0 ± 9.1	93.9 ± 8.2
No	701	63.5	71.4 ± 7.2	87.4 ± 9.7

**Table 2:** Results of logistic regression analysis for individual factors.

	OR	95% CI	*p*-Value
Gender			
Male vs. female	1.86	(1.46, 2.37)	<0.001
Age (ref: less than 21 years old)			
21–30 years old	5.05	(2.77, 9.21)	<0.001
31–40 years old	3.20	(1.75, 5.84)	<0.001
41–50 years old	1.28	(0.71, 2.29)	0.412
51–60 years old	1.42	(0.78, 2.61)	0.253
61 years old and above	0.61	(0.27, 1.36)	0.226
Education (ref: secondary school and below)			
Bachelor	2.18	(1.46, 3.26)	<0.001
Master	1.55	(1.02, 2.36)	0.039
Ph.D.	19.57	(6.59, 58.15)	<0.001
Received radiation training			
Yes vs. no	14.52	(9.84, 21.43)	<0.001
Radiation related job			
Yes vs. no	10.70	(7.88, 14.52)	<0.001

**Table 3:** The *t*-test results of received radiation education and engaged radiation-elated job within each age subgroup.

	Received Radiation Education		Engaged Radiation-Related Job	
	Knowledge Index (Mean ± SD)	*p*-Value	Knowledge Index (Mean ± SD)	*p*-Value
Less than 21 years old				
Yes	81.3 ± 8.3	<0.001	87.6 ± 8.5	<0.001
No	70.1 ± 5.6		70.5 ± 5.8	
21–30 years old				
Yes	86.1 ± 8.5	<0.001	83.7 ± 9.2	<0.001
No	76.0 ± 9.2		73.7 ± 8.8	
31–40 years old				
Yes	86.0 ± 8.0	<0.001	83.1 ± 9.5	<0.001
No	74.0 ± 9.0		72.1 ± 8.0	
41–50 years old				
Yes	81.3 ± 8.6	0.001	80.4 ± 8.4	<0.001
No	71.1 ± 6.8		71.4 ± 7.2	
51–60 years old				
Yes	83.8 ± 8.2	<0.001	83.9 ± 8.7	<0.001
No	71.0 ± 6.5		71.1 ± 6.5	
61 years old and above				
Yes	78.1 ± 4.2	0.002	81.3 ± 4.8	<0.001
No	69.4 ± 5.9		87.6 ± 8.5	

**Table 4:** The *t*-test results of received radiation education and engaged radiation-elated job within each education subgroup.

	Received Radiation Education		Engaged Radiation-Related Job	
	Knowledge Index (Mean ± SD)	*p*-Value	Knowledge Index (Mean ± SD)	*p*-Value
Secondary school and below				
Yes	81.4 ± 9.1	<0.001	78.9 ± 10.1	<0.001
No	69.2 ± 6.4		69.0 ± 6.0	
Bachelor				
Yes	84.9 ± 8.5	<0.001	82.8 ± 9.1	<0.001
No	73.1 ± 8.5		71.4 ± 7.4	
Master				
Yes	84.0 ± 8.8	<0.001	83.8 ± 8.8	<0.001
No	71.9 ± 7.0		71.8 ± 7.0	
Ph.D.				
Yes	87.4 ± 6.0	<0.001	87.1 ± 6.5	<0.001
No	77.8 ± 6.7		78.4 ± 6.8	

## Data Availability

The data presented in this study are available on request from the corresponding author. The data are not publicly available due to the protection of detail raw data.

## Supplementary Materials

The following supporting information can be downloaded at: https://www.mdpi.com/article/10.3390/ijerph192013422/s1, Supplementary File S1: Questions of the survey.
