# Supplementary material for: The Analysis with Quantitative Indexes for Public’s Awareness of Radiation Knowledge in Taiwan
Source: Int J Environ Res Public Health. 2022 Oct 17;19(20):13422. doi: 10.3390/ijerph192013422 (PMC9603622; doi:10.3390/ijerph192013422)
Supplement: Supplementary file 1 [file ijerph-19-13422-s001.zip › ijerph-1941895-supplementary.pdf]

Supplementary File S1 - Questions of the survey

First section - personal information of survey respondents

1. What is your gender?
   Male
   Female
2. What is your age?
   Less than 21 years old
   21 - 30 years old
   31 - 40 years old
   41 - 50 years old
   51 - 60 years old
   61 years old and above
3. What is your education level?
   Secondary school and below
   Bachelor
   Master
   Ph.D
4. What is your job?
   Student
   Civil servant
   Professional
   Financial industry
   Information technology
   Service and sale
   Communication and culture
   Manufacturing
   Construction
   Agriculture, forestry, fisheries and animal husbandry
   Freelance
5. Have you attended any radiation education training?
   Yes
   No
6. Do you engage in radiation-related job?
   Yes
   No

Second section - questions about radiation knowledge and education responsibility

The following table listed the questions and the corresponded average scores. The survey was designed basing on Likert scale. The score of each question would be converted basing on five choices to response: very agree, agree, no opinion, disagree, very disagree. The number in parentheses after question was the full score.

Table S1. Basic knowledge of radiation

	Average score
Radiation is a form of energy transfer. (5)	4.6 ± 0.6
Sunlight, visible light, and X-rays are all radiation. (5)	4.5 ± 0.8
Human body can sense the presence of radiation through receptors. (5)	3.2 ± 1.3
Food will be radioactive if it is exposed to radiation. (5)	2.9 ± 14
Radiation is widely used in civil, medical, industrial and defense fields. (5)	4.5 ± 0.6

Table S2. Environmental radiation

	Average score
The radiation dose received on high mountain is lower than that on the ground. (5)	3.3 ± 1.3
Drinking water, fruits and vegetables are all contain environmental radiation. (5)	4.1 ± 0.9
There are many kinds of radionuclides in soil. (5)	4.2 ± 0.8
Smoking will make people receive radiation dose. (5)	3.4 ± 1.2
Under normal conditions, flight attendants have higher environmental radiation doses than factory workers. (5)	4.0 ± 1.0

Table S3. Medical radiation.

	Average score
Do you think that medical radiation can benefit human body? (5)	3.0 ± 1.2
After taking X-ray in hospital, you can't touch the wall of examination room since there is radiation residue. (5)	3.3 ± 1.2
Human body will be radioactive after taking X-ray examination. (5)	3.4 ± 1.2
Both computer tomography (CT) and magnetic resonance imaging (MRI) use radiation for their examination. (5)	2.5 ± 1.3
Cancer will definitely occur after exposure to radiation. (5)	3.9 ± 1.0

Table S4. Radiation science

	Average score
Radiation can be used for non-destructive testing. (5)	4.3 ± 0.7
Radiation has no benefit to living organisms. (5)	3.7 ± 1.1
Hokutolite is a radioactive mineral. (5)	3.9 ± 0.9
The unit used to assess radiation dose is 'sievert (Sv)'. (5)	4.1 ± 0.9
The annual dose limit of radiation workers is higher than that of general public. (5)	3.3 ± 1.4

Table S5. Radiation protection

	Average score
During X-ray examinations, lead apron can be worn to reduce radiation dose outside the examination area. (5)	4.2 ± 1.0
The longer you are in contact with the radiation source, the higher the radiation dose you will receive. (5)	4.4 ± 0.7
The farther away you are from the radiation source, the higher the radiation dose you will receive. (5)	3.7 ± 1.3
Taking iodine tablets can protect thyroid if radiation leaks from nuclear power plant. (5)	3.8 ± 1.0
The radiation control agency in our region is the Atomic Energy Commission.(5)	4.4 ± 0.7

Table S6. Social responsibility for radiation awareness

	Average score
The following pictures is radiation warning sign. (5) 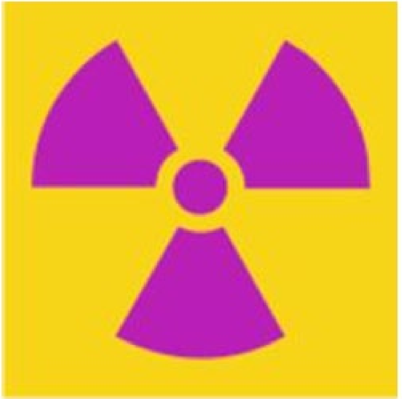	4.5 ± 0.7
Do you think radiation is everywhere? (5)	4.5 ± 0.6
Do you think the correct use of radiation is beneficial to humans? (5)	4.3 ± 0.8
Do you think radiation education is an important issue? (5)	4.6 ± 0.6
Do you think universities need to promote the correct awareness of radiation knowledge? (5)	4.6 ± 0.6
